# Refracture, a Potential Complication of the Periosteal Flap Technique Without Bone Graft for Isolated Congenital Pseudarthrosis of the Fibula: A Report of Two Cases and Literature Review

**DOI:** 10.5435/JAAOSGlobal-D-22-00046

**Published:** 2023-06-12

**Authors:** Ramin Zargarbashi, Mazaher Ebrahimian, Salar Baghbabi, Alireza Moharrami, Younes Basti

**Affiliations:** From the Department of Orthopedic Surgery, Tehran University of Medical Sciences, Tehran, Iran (Dr. Zargarbashi), Department of Orthopedic Surgery, Sina Hospital, Tehran University of Medical Sciences, Tehran, Iran (Dr. Baghban), Joint Reconstruction Research Center, Tehran University of Medical sciences, Tehran, Iran (Dr. Moharrami), Department of Orthopedic Surgery, Sina Hospital, Tehran University of Medical Sciences, Tehran, Iran (Dr. Basti).

## Abstract

It is a rare condition defined by congenital pseudarthrosis of the fibula (CPF), which may be associated with neurofibromatosis (NF) and congenital pseudarthrosis of the tibia. A condition such as this may be symptomatic or asymptomatic, but it requires treatment because of the progression of signs such as ankle valgus and pseudarthrosis of the tibia. There are a number of surgical techniques that can be used to treat this condition, such as tibiofibular fusion, internal fixation, the Ilizarov method, and periosteal flap.

**Aim:**

The goal of this study was to describe the treatment results of two patients with CPF using vascularized fibular periosteal flaps.

**Case report:**

We described the case of a 5-year-old patient and a 19-month-old patient with isolated CPF. Both patients underwent a distal-based vascularized fibular periosteal flap, and intramedullary fixation was used to treat the patients.

**Conclusion:**

The patients had full union in the pseudarthrosis site, but in the end, both had asymptomatic refracture in the union site. Our experiences showed that it is necessary to use strong intramedullary fixation and bone graft.

Congenital pseudarthrosis of the fibula (CPF) is a rare condition that can be associated with neurofibromatosis (NF) or congenital pseudarthrosis of the tibia (CPT).^1,2^ Approximately 50% of CPF cases are associated with NF, but it is not known how these diseases contribute to CPF.^2,3,4,5^ The etiology and pathology of CPF and bowing are the same as for CPT and anterior bowing.^6^ CPF has a wide range of symptoms, from bowing/loss of corticomedullary to fractures, to pseudarthrosis, to significant bone loss.^7^ Using the type of CPF presentation, Dooley et al^6^ proposed a classification in four parts and described the types of CPF.

Specifically, Dooley et al^6^ classified the condition as follows: (1) pure fibular bowing without pseudarthrosis, (2) fibular pseudarthrosis without ankle deformity, (3) fibular pseudarthrosis with ankle deformity, but not late development of tibial pseudarthrosis, and (4) fibular pseudarthrosis with late development of tibial pseudarthrosis. The classification of CPT by Boyd and Andersen^8^ includes types 1 and 4^9^ while types 2 and 3 are considered to be isolated CPFs.^10^ CPF has been controversially treated in the literature; Dooley et al. recommended distal tibiofibular fusion because of the risk of failed fibular osteosynthesis and progressive ankle valgus.^6,11^ Contrarily, the study by Martus and Johnston^9^ found no ankle valgus in their cases and recommended against fusion of the tibia and fibula. Consequently, CPF can lead to ankle valgus and instability in the weight-bearing joint and joint osteoarthritis,^1,12^ and for these reasons, many surgical techniques have been proposed.

Therefore, treatment of CPF is required, and it should be done as soon as a progressive aggravation of ankle valgus is noted.^1^ Studies dedicated to CPF are rare in the literature. Because of the rarity of this disease and the difficulty that this pathology presents, the proposed treatments are numerous and different.^3,6,9,11,12^ The periosteum is an important part of the child's skeleton and has great osteogenic power, so we studied periosteal flap surgery as a method for treating CPF. There were periosteal vascularized tissues capable of generating bone at the site of pseudarthrosis.^13,14^ Recent studies show that vascularized fibular periosteal flap (VFPG) transfers are effective for treating bone-healing problems in children. There is a strong possibility that it has osteogenic and angiogenesis potential because of its cambium layer being a source of stem cells.^14^ In 2010, Trigui et al^15^ treated two patients with CPF using the VFPG technique and recommended it for young children with CPF. In this study, we describe the results of the two cases of CFP treated with vascularized fibrous periosteal flaps.

## Case Report

### Case 1

A 19-month-old boy referred to our clinic with a fibula distal shaft fracture was diagnosed at 12 months, but was asymptomatic at the time. At the time, neither his ankles nor his feet showed any deformity. No NF was noted in the family, and the spinal examination was normal. He did not have any spots of cafe-au-lait staining on his body. The patient was able to walk with full weight bearing and was free of pain or tenderness.

**Figure 1:**
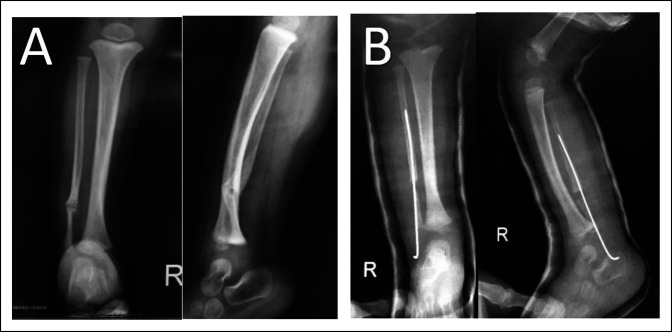
A 9-month-old boy with congenital pseudarthrosis (congenital pseudarthrosis of the fibula) of the fibula type 3 Dooley (**A**), as seen in the postoperative radiograph. A transferred periosteal flap technique was conducted for the patient (**B**).

A radiograph of the right ankle and leg showed CPF in the lower third of the bone with no deformity of the tibia or ankle. According to Dooley et al (fibular pseudarthrosis without ankle deformity), the patient was classified as a type 2 CPF with 88° in the lateral distal tibial angle (Figure 1A).

**Figure 2:**
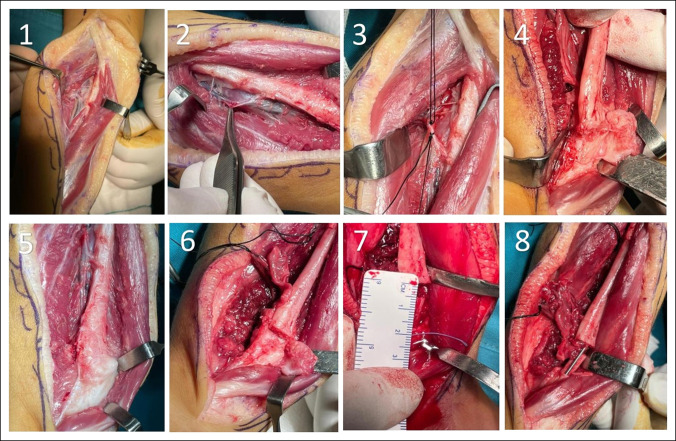
A transferred periosteal flap technique was conducted for the patient with the distal base pedicle (1-8).

VFPG was recommended for this case, based on some studies. We conducted a lateral incision for the patient, cleared the fracture site of soft tissues and callus, and left a 2-cm gap in the fracture site. VFPG was transferred from the distal end of the fibula and sutured around the fracture site (Figure 2). In addition, we used a 1.5-mm intramedullary Rush nail to stabilize the fracture site (Figure 1B).

**Figure 3:**
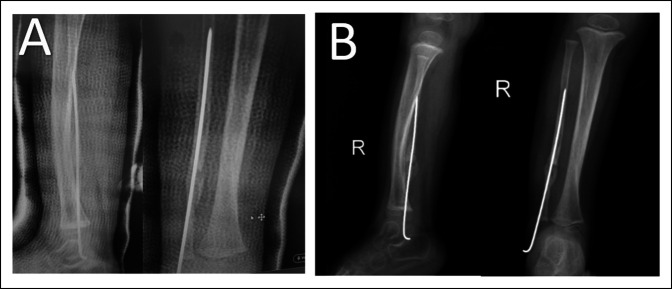
After 4 weeks, we saw callus formation in the radiograph (**A**); in the 3-month postoperative radiograph, we saw full union in the congenital pseudarthrosis of the fibula site, and we extracted the Rush nail from the bone (**B**).

**Figure 4:**
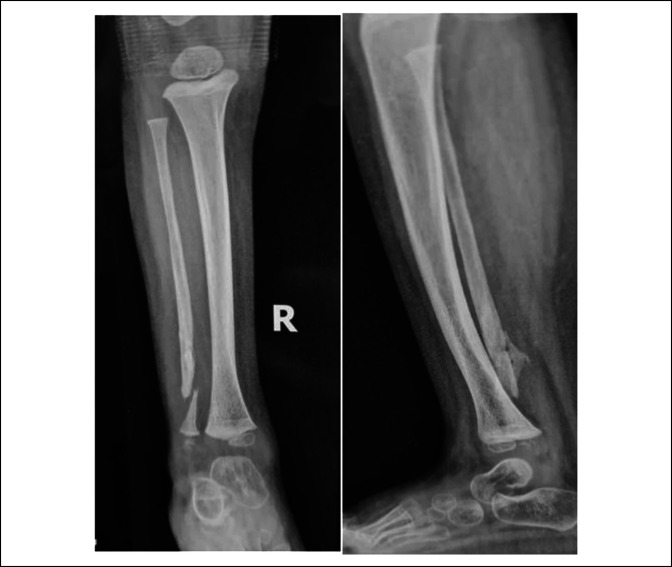
Nine months after surgery, we saw asymptomatic refracture in the union site.

Callus formation was seen 4 weeks after surgery (Figure 3A). We removed the Rush nail from the bone when we saw full callus formation on the leg radiograph after 12 weeks (Figure 3B). Radiographs were obtained after 3 months, and we started full weight bearing for the patient. The patient was followed for 9 months without pain, tenderness, or limping. We noted an asymptomatic refracture in the pseudarthrosis site of the fibula after the 9-month follow-up visit (Figure 4). The complication was managed conservatively without surgery because no symptoms were present.

### Case 2

A 5-year-old boy with NF referred to our clinic complaining of ankle deformity, limping, and pain. In his 2-year-old age, the patient had an ankle deformity and distal tibia and fibular bowing that were classified as type 1 according to the Dooley classification of fibula pseudarthrosis. A distal tibial hemiepiphysiodesis of the anterolateral distal tibia was conducted 3 years before referral to our center with fracture in the fibula.

**Figure 5:**
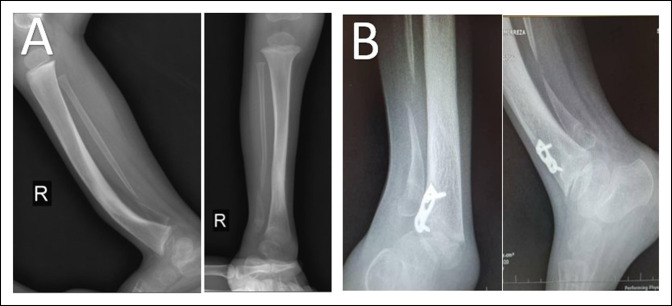
A 5-year-old boy with congenital pseudarthrosis (congenital pseudarthrosis of the fibula) of the fibula type 3 Dooley (**A**), as seen in the postoperative radiograph 2 years before referring. An anterolateral hemiepiphysiodesis was conducted for the patient (**B**).

The patient was referred to our center when the leg and ankle radiographs showed a pseudarthrosis of his distal fibula and ankle (tibia) valgus, which was classified as a type 3 with 72° in the lateral distal tibial angle according to the CPF classification by Dooley et al^6^ (Figure 5).

**Figure 6:**
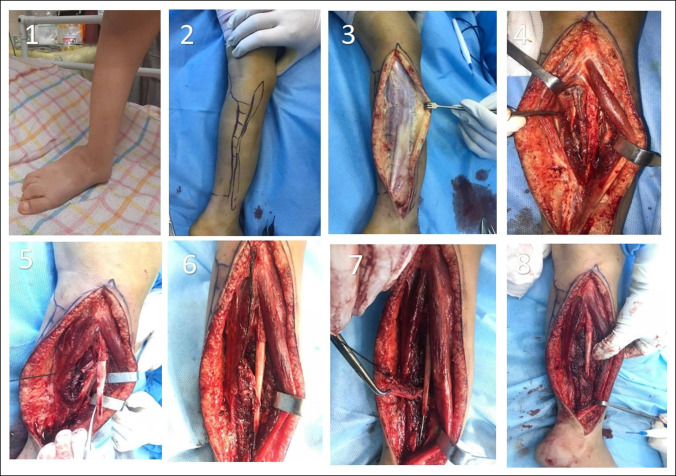
A transferred periosteal flap technique and anteromedial hemiepiphysiodesis were conducted for the patient with the distal base pedicle (1-8).

**Figure 7:**
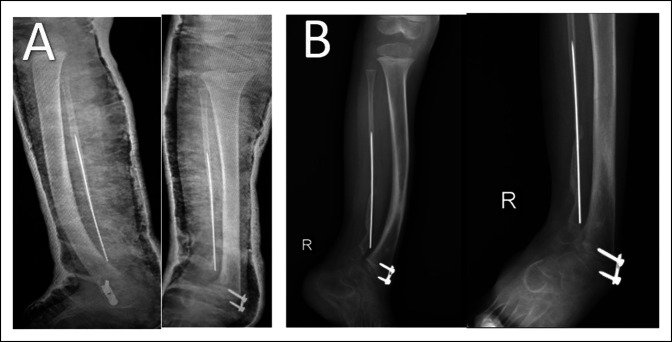
After 4 weeks, we saw callus formation in the radiograph (**A**); in the 3-month postoperative radiograph, we saw full union in the congenital pseudarthrosis of the fibula site (**B**).

The patient was the same as the previous case, and he was eligible for VFPG. We conducted the lateral incision on the patient and then removed the soft tissues and callus from the fracture site. We stabilized the fracture site primarily through the passage of a 1.5-mm intramedullary Rush nail, leaving a 2-cm gap at the fracture site. Transfer of the VFPG from the fibula distal end was done, and sutures were placed around the fracture site (Figure 6). From the lateral approach, we also extracted the 8-plate hemiepiphysiodesis. We conducted an anteromedial distal tibia hemiepiphysiodesis with a medial approach using a 2-hole 3.5-mm reconstruction plate for the patient because of distal tibia deformity (Figure 7A).

**Figure 8:**
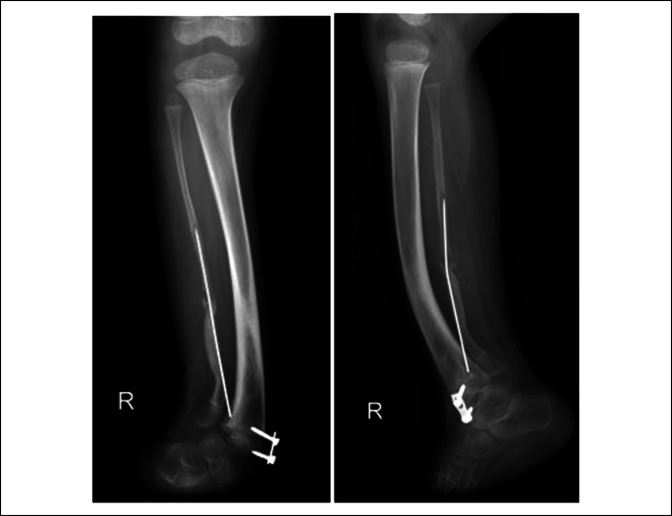
Nine months after surgery, we saw asymptomatic refracture in the union site.

The patient had full weight bearing when we saw full callus formation in the leg radiograph (Figure 7B). Three months after surgery, the radiographs showed full union of the fibula, and the patient was followed for 9 months with no complications, including pain, tenderness, and limping. We found that an asymptomatic refracture had occurred in the pseudarthrosis site of the fibula in the 9-month follow-up visit (Figure 8). The complication was also treated conservatively without surgery because of the absence of any symptoms.

## Technique of Distal Pedicled Returned Periosteal Flap

The periosteal flap technique for CPF was described by Trigui et al.^15^ As the patient lies supine with a raised homolateral buttock, the air tourniquet is applied. We also used a lateral approach to access the fibula. During surgery on the fibula, an incision is made from the superior third of the bone to the lateral malleolus. A fascia superficialis incision is made; the peroneus longus and brevis muscles and the musculocutaneous nerve (superficial peroneal nerve) are spread forward from the fibula while keeping 1 to 2 mm of muscle insertion on the fibula. After opening both lateral and superficial posterior compartments, the soleus is retracted to the posterior, and the interosseous membrane is opened vertically close to the bone. As a result, the fibular artery and vein are exposed and get near the periosteum. The lower third of the leg is also dissected to separate the fibular pedicle from the pseudarthrosis site. Then, we incision the periosteum and cleave it from the lateral face of the diaphysis and then proximal end circumferential periosteal incision was made (a tube of periosteum attached to distal). Close to the posterior tibial vessels, the proximal end of the vessels was ligated and sectioned; therefore, we get a fibular periosteal flap pedicled to a distal vessel, and also the flap is raised proximal to the pseudarthrosis site. As a rule of thumb, the lower limit of the flap should be within two to three centimeters of the pseudarthrosis site to ensure that the flap is obtained from healthy tissues entirely. Afterward, the fibular flap is sutured to the distal fibular metaphysis, the proximal part of the flap being sutured to the outer surface (outer cortex), and the rest of the flap is attached to the inside of the metaphysis (inner cortex) as a tube. Finally, we checked the pedicle flow with intraoperative Doppler sonography. A closure is made after the tourniquet has been loosened and the hemostasis and drainage have been confirmed (Figures 2 and 6). The immobilization is accomplished by plastering the area.

## Discussion

There is a rare condition known as CPF that is associated with NF and CPT.^1,2^ Nearly 50% of CPF cases are associated with NF; the contributing mechanism is unknown.^3-5^ In light of the important role played by the periosteum in children and its osteogenic potential, we studied the treatment of CPF using periosteal flaps. The pseudarthrosis was mainly composed of a periosteal vascularized tissue capable of replicating bone. A VFPG transfer for treating challenging bone healing problems in children has recently been described.^14,15^ Our study presents two cases of CPF with different types of CPF, which were treated with distal base fibular periosteal flap without any bone grafting.

In our first case, we conducted fibula vascularized periosteal flap surgery on a 9-month-old patient with Dooley type 2 of CPF. Three months after surgery, the patient had completely healed, with no need for bone grafting in the 2-cm gap. According to Trigui et al,^15^ the technique produced a good outcome with a full union while no follow-up results were available. We followed our patient for 9 months without any complications, but after 9 months, we discovered that she had asymptomatic refracture at the union site. In this case, we treated the refracture with conservative treatment, but we did not follow up with the patient after refracture. We suggest using a permanent intramedullary device in this case without extraction and perhaps bone grafting in the surgical technique, based on our experience.

In our second case, a 5-year-old boy with NF underwent surgery 2 years ago because of CPF Dooley type 3. The patient underwent an anterolateral distal tibia hemiepiphysiodesis three years before referral to our center. We conducted vascularized fibula periosteal flap and also extracted the anterolateral plate and replaced it with an anteromedial plate for hemiepiphysiodesis on the patient. The patient was followed for 9 months with full union and increased LDTAs. We saw asymptomatic refracture of the fibula in the union site after 2 years, as we did with the first patient. As in this case, we had the same complication, and we think that using stronger intramedullary fixation or internal fixation for patients treated with this technique may prove helpful in preventing refracture and using bone graft. The technique has been used to treat two patients with CPF with a 2-cm gap in the pseudarthrosis site without the need for bone grafts, resulting in full union after 12 weeks in both patients, but we say a refracture in the union site. Based on our experiences in these cases, this technique needs bone grafting and permanent strong intramedullary fixation. In previous studies, different treatment methods for this condition were recommended, but long-term outcomes were not reported.^1-3,6,7,9,10,12,15^

In this study, we reviewed the literature and all the studies that reported isolated congenital fibula pseudarthrosis as presented in Table 1. According to Dooley et al. in 1974^6^ and Dal Monte et al. in 1987,^16^ fusion of the tibia and fibula produced good results, but Merkel et al. reported an 11-month case of isolated CPF treated with open-wedge valgus osteotomy and casting in 1984.^3^ In the patients with nonunion of the fibula, osteosynthesis with an iliac corticocancellous graft was conducted 9 months postoperatively, and full union was obtained after 4 years of follow-up.^3^ Two cases of isolated CPF were reported in 2019;^12^ the first was a 13-year-old adolescent boy treated with osteosynthesis and internal fixation with a good outcome for 2 years without any complications. The second patient had Dooley type 3 of CPF, which was treated by tibial frontal osteotomy, distal tibiofibular fusion, and medial malleolus epiphysiodesis. In this patient, the clinical and radiologic outcomes were favorable over a period of 4 years.^12^

**Table 1:** The Table Shows That the Studies Reported About Isolated Congenital Fibula Pseudarthrosis

Author	Year	N	Age	NF	Dooley Grade	Treatment	Delay Healing	Follow-up	Results
Dooley et al	1974	4	11 mos	+	1	Close follow-up	NR	37 mos	Fibular bowing progression
			15 mos	−	2	Close follow-up	NR	84 mos	Persist pseudarthrosis
			12 yrs	—	3	Supramalleolar osteotomy + distal fixed by 2 screws	3 mos	12 mos	Normal gait and anatomy
			13 mos	−	4	Intramedullary nailing + autograft	4 mos	48 mos	Tibial bowing and fracture
Merkel et al	1984	1	11 mos	—	1	Tibial open-wedge osteotomy + fibula oblique osteotomy	4 mos	72 mos	Ankle valgus deformity + persist fibular pseudarthrosis
Dal Monte et al	1987	3	20 mos	−	3	Autografting + casting	NR	36 mos	Ankle valgus deformity + persist fibular pseudarthrosis
			3 yrs	+	2	Close follow-up	NR	9 yrs	Normal gait and anatomy
			8 yrs	+	1	Tibiofibular osteodesis + autograft of the fibula	3 mos	48 mos	Ankle valgus deformity + persist fibular pseudarthrosis
DiGiovanni et al	1998	1	11 yrs	+	3	Interpositional allograft + cross Kirschner wires + medial distal tibia hemiepiphysiodesis	5 mos	42 mos	LLD (leg-length disappearance) + persist fibular pseudarthrosis
Yang et al	2002	3	6 mos	+	3	Close follow-up	NR	16 mos	Normal gait and anatomy
			14 mos	−	2	Close follow-up + ankle-foot orthosis	NR	28 mos	Normal gait and anatomy
			18 mos	+	2	Tibia-fibula syndesmosis fusion + CFP autograft	4 mos	30 mos	Normal gait and anatomy
Lampasi et al	2008	2	2.5 yrs	−	2	Distal tibia-fibula fusion and subtalar screw arthroereisis + autograft	3 mos	36 mos	Normal gait and anatomy
			18 mos	−	3	Intramedullary nailing + autograft	NR	52 mos	Persist fibular pseudarthrosis
Trigui et al	2010	6	5.5 yrs	+	3	Free periosteal flap	18 mos	14 yrs	Ankle valgus
			3 yrs	+	3	Distal pedicle periosteal flap	3.5 mos	3.5 yrs	Normal gait and anatomy
			3.5 yrs	+	3	Proximal pedicle periosteal flap + spongious graft-screwed plate	12 mos	8 yrs	Normal gait and anatomy
			4 yrs	+	3	Proximal pedicle periosteal flap + spongious graft-screwed plate	NR	1.5 yr	Normal gait and anatomy
			5.5 yrs	+	4	Distal pedicle periosteal flap + intramedullary wire	10 mos	1 yr	Normal gait and anatomy
			3 yrs	+	4	Distal pedicle periosteal flap + intramedullary wire	NR	1 yr	Ankle valgus
Cherrad et al	2019	2	13 yrs	−	3	Osteosynthesis of the fibula with plate + autograft + epiphysiodesis of the distal tibia	6 mos	24 mos	Normal gait and anatomy
			12 yrs	−	3	Distal tibia-fibula fusion + frontal osteotomy of the tibia + epiphysiodesis of the medial malleolus	2 mos	48 mos	Normal gait and anatomy
Wang et al	2020	15	Median 3.8 yrs	12 +	1 (7 pts) 2(4 pts) 3 (3 pts) 4(1 pt)	Fibular osteosynthesis (2 pts) Distal tibia-fibula fusion (10 pts) Medial hemiepiphysiodesis (1 pt) Below-knee amputation (1 pt)	NR	11.8 yr	Failed fibular osteosynthesis (4 pts) converted to tibia-fibula fusion Refracture (1 pt) and bone grafting Normal gait and anatomy (10 pts)
Our study	2022	2	19 mos	−	2	Distal pedicle periosteal flap + intramedullary wire	3 mos	9 mos	Asymptomatic refracture of the fibula
			5 yr	+	3	Distal pedicle periosteal flap + intramedullary wire + medial distal tibia hemiepiphysiodesis	3 mos	9 mos	Asymptomatic refracture of the fibula

NF = neurofibromatosis, NR = not reported

An important aspect of our study is the use of the periosteal flap technique without any bone grafts or permanent fixation devices. On radiographic evaluation, we found full union, and the patients did not complain of any pain or limping. In our cases, we observed refracture of the union site; however, this was not symptomatic. Consequently, we managed the complication conservatively. However, this is the second study to use this technique for CPF. The study by Trigui et al^15^ reported good results for six patients with this technique, but they used bone graft for two patients and permanent intramedullary wire for two patients. In addition, their follow-up for the patients with distal pedicled flap was short. According to our experience, this method should be used with permanent intramedullary fixation to avoid refracture of the union site.

## Conclusion

It is a preferred option in CPF treatment of bony defects with full union by using bone graft in our 2 cases. We found that this technique requires a strong intramedullary fixation with Kirschner wire or Rush nail and bone grafting to prevent complications such as refracture at the union site. However, more cases and larger studies are needed to provide definitive results in the future.
